# DeadEasy Caspase: Automatic Counting of Apoptotic Cells in Drosophila

**DOI:** 10.1371/journal.pone.0005441

**Published:** 2009-05-05

**Authors:** Manuel G. Forero, Jenny A. Pennack, Anabel R. Learte, Alicia Hidalgo

**Affiliations:** NeuroDevelopment Group, School of Biosciences, University of Birmingham, Birmingham, United Kingdom; Institute for Research in Biomedicine, Spain

## Abstract

Development, cancer, neurodegenerative and demyelinating diseases, injury, and stem cell manipulations are characterised by alterations in cell number. Research into development, disease, and the effects of drugs require cell number counts. These are generally indirect estimates, because counting cells in an animal or organ is paradoxically difficult, as well as being tedious and unmanageable. Drosophila is a powerful model organism used to investigate the genetic bases of development and disease. There are Drosophila models for multiple neurodegenerative diseases, characterised by an increase in cell death. However, a fast, reliable, and accurate way to count the number of dying cells in vivo is not available. Here, we present a method based on image filtering and mathematical morphology techniques, to count automatically the number of dying cells in intact fruit-fly embryos. We call the resulting programme DeadEasy Caspase. It has been validated for Drosophila and we present examples of its power to address biological questions. Quantification is automatic, accurate, objective, and very fast. DeadEasy Caspase will be freely available as an ImageJ plug-in, and it can be modified for use in other sample types. It is of interest to the Drosophila and wider biomedical communities. DeadEasy Caspase is a powerful tool for the analysis of cell survival and cell death in development and in disease, such as neurodegenerative diseases and ageing. Combined with the power of Drosophila genetics, DeadEasy expands the tools that enable the use of Drosophila to analyse gene function, model disease and test drugs in the intact nervous system and whole animal.

## Introduction

The quantitative analysis of cell death (apoptosis) is required to solve fundamental questions of developmental biology and to understand disease. Multiple disease conditions result from alterations in the control of cell survival, most notoriously neurodegeneration (e.g. Alzheimer's and Parkinson's diseases, and demyelinating diseases such as multiple sclerosis). Injury (e.g. spinal cord injury) results in an increase in cell death, plus a homeostatic regulation of cell proliferation. Drosophila is a very powerful model organism that has led to the discovery of gene networks and gene functions involved in development, cancer and neurodegeneration [1]. There are Drosophila models for Alzheimer's and Parkinson's disease, spongiform disease and several ataxias. However, alterations in cell number can be missed by conventional phenotypic approaches that do not inspect cell number. Approaches that estimate cell number based on general properties (e.g. anatomy, volume or area covered by pixels) miss phenotypes in cell number that are subtle, that do not lead to anatomically visible consequences or that affect a fraction of cells (i.e. specific cell types). To further exploit the power of Drosophila for the genetic analysis of cell number control, accurate methods to quantify apoptosis are needed.

Functional analyses of molecules controlling cell number have frequently been carried out in cell culture or after cell dissociation; counting cells in the intact animal (i.e. in vivo) is generally carried out manually, and may consist of estimates of number of cells stained with a particular cell marker or inferences from anatomical alterations [2]–[5]. In Drosophila, manual counting, throughout the nervous system, is carried out using antibodies to visualise particular cell states or cell lineages [6]–[11]. While these methods are most accurate and most appropriate for certain questions, they can be extremely time-consuming and/or inappropriate for other questions. There was a compelling case for developing automatic cell counting software.

Computational quantification in intact animals requires object recognition solutions in 3D, which can be achieved with confocal microscopy. Most available software programmes for automatic cell counting are not applicable in vivo because they either estimate cell mass (e.g. FACS based), or apply image processing and pattern recognition in 2D (e.g. Metamorph). Projection of all images of a confocal stack into one single 2D image is not appropriate. Automatic techniques have been developed to segment cell nuclei from tissue sections or whole Drosophila brains in 2D and 3D images [12]–[15], but they require intensive computation, making them unsuitable for large sample sizes. A simple method for estimating areas occupied by Caspase-stained pixels of intensity over an empirical threshold has been used to get an “apoptotic index”, but this method does not count cells [16]. Cell profiler software [17] allows users to combine image-processing methods to develop techniques to count cells, but this requires some knowledge of computation and it has not been tested for specific applications. Thus, as yet, no automatic, fast and easy method to count different cell types or cell states (i.e. apoptosis) in vivo exists.

Here, we have developed a method for automatic quantification of dying cells in the intact animal Drosophila embryo. In companion papers, we will report methods for automatic counting of mitotic cells, neurons and glia. DeadEasy Caspase counts apoptotic cells and has been validated for a range of specified parameters in Drosophila. DeadEasy Caspase has been written in Java as a freely available ImageJ plug-in, and the programme is accessible for further creative modifications by researchers beyond the Drosophila community and within the general biomedical community.

## Materials and Methods

### Mathematical algorithm for object recognition

**Figure 1:**
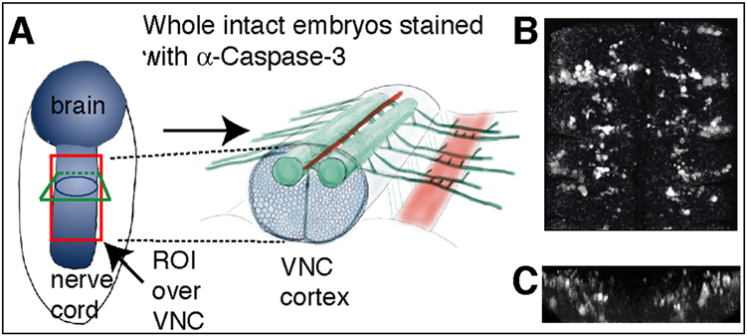
The extent of apoptosis in the Drosophila embryonic VNC. (A) A Drosophila embryonic VNC. The Region Of Interest (ROI) box in red indicates the areas analysed in (B,C). (B) 3D rendering of confocal stacks of sections through the VNC to show the abundant number of embryonic apoptotic cells stained with anti-cleaved-Caspase-3. (C) Cross section view of the image in (B).

**Figure 2:**
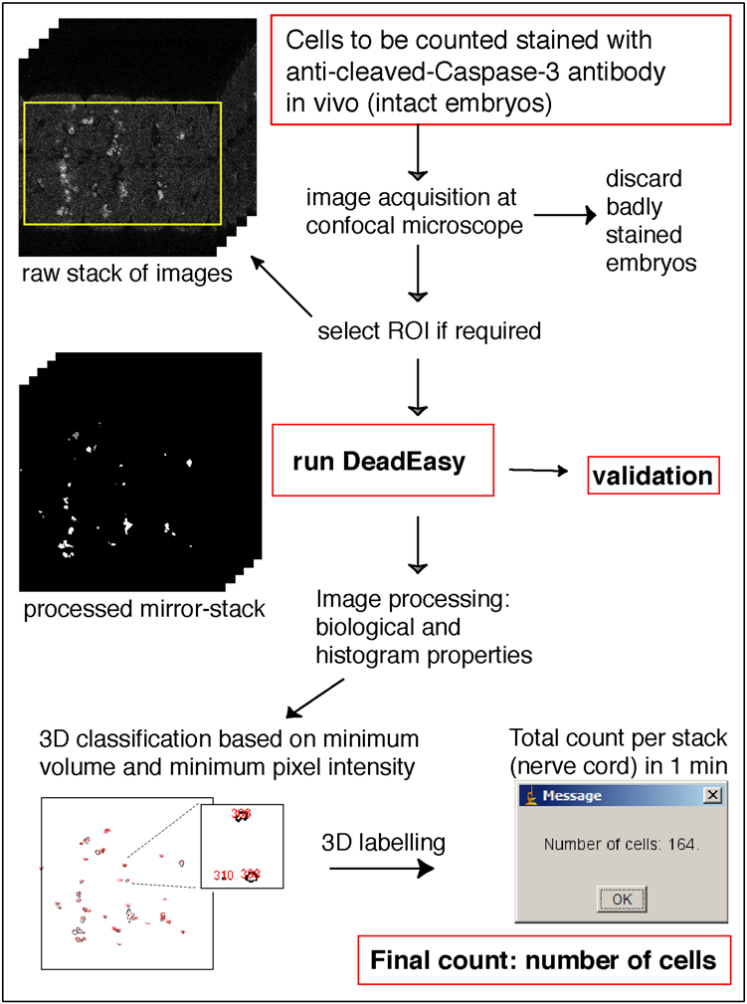
How DeadEasy software works.

**Figure 3:**
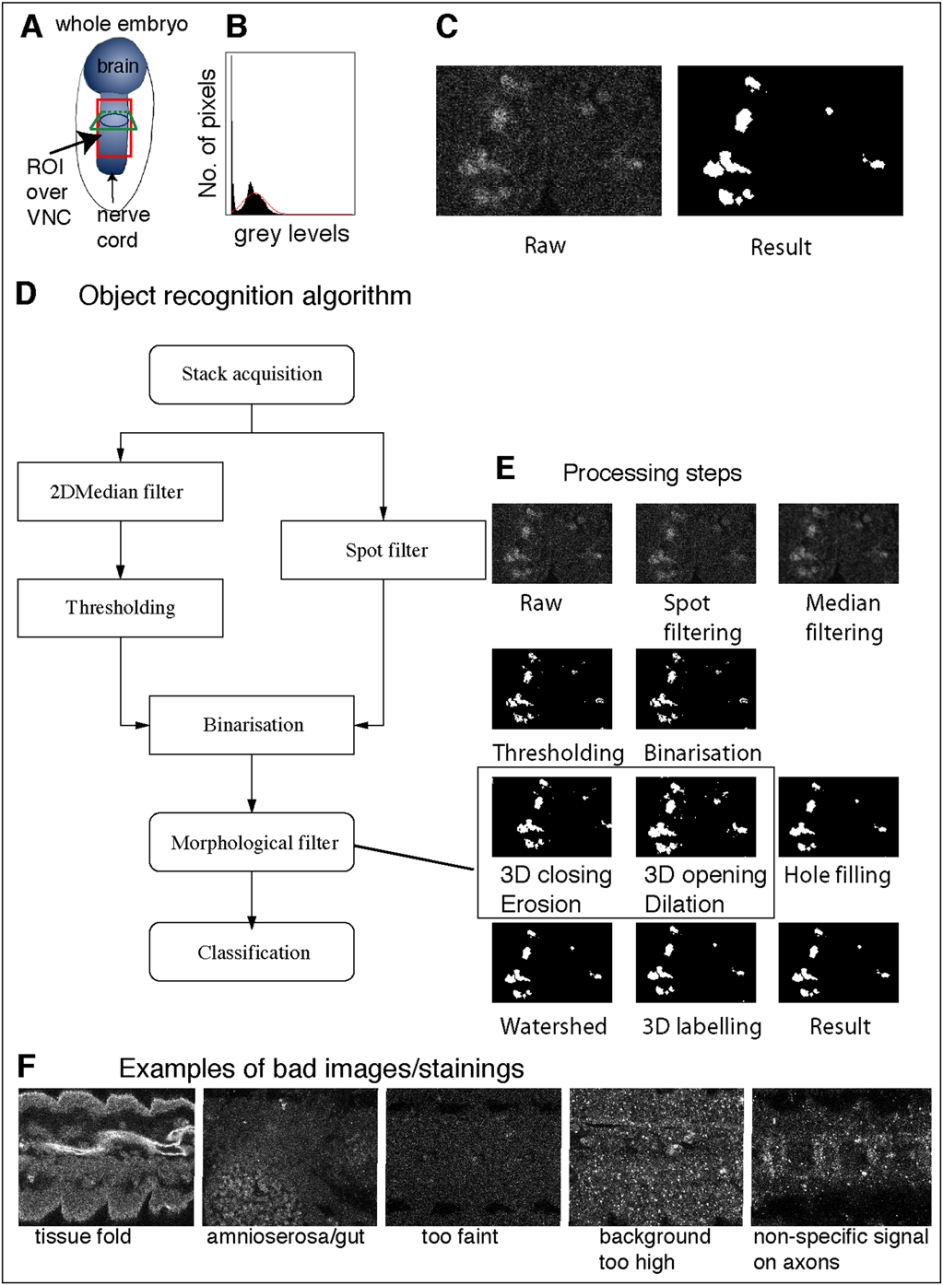
DeadEasy Caspase: the mathematical algorithm. DeadEasy Caspase detects apoptotic cells in embryos stained with anti-cleaved-Caspase-3, and are characterised by low cytoplasmic signal, high background and volume ≥1.56 µm^3^. (A) Diagram showing the region of the embryonic VNC (blue) scanned for counting. (B) Caspase histogram. (C) Enlarged images to compare a raw image vs. the result (single confocal 0.25 µm slices shown). (D) Diagram of the algorithm. (E) Images showing the different steps of processing, correlating with each step in the diagram in (D). (F) Examples of bad quality stainings or images that must not be used for cell counting as they will produce false positives.

Intact whole embryos are fixed and stained with Caspase antibodies. A stack of around 150 slices per specimen is acquired by laser-scanning confocal microscopy throughout the thickness of the nerve cord (Figure 1). Counting can be confined to a Region Of Interest (ROI). In confocal microscopy images, noise follows a Poisson distribution due to the fact that image acquisition is based on photon detection. Several noise reduction techniques based on wavelets can be employed to filter the images. These can yield good results with an appropriate bank of filters. Instead, we used a median filter, because it is the simplest method and it was found to give good results. This reduces the noise and the significant intensity heterogeneity typical of confocal images, without strongly affecting the signal provided by the stained cells. After filtering, segmentation is carried out. To process a stack of images, 3D image processing techniques can be applied to improve the quality of segmentation [18], but working in 3D is challenging. In confocal microscopy, photo-bleaching and florescence attenuation with increasing depth by finite sample transparency (related to sample thickness), result in signal intensity decaying with increasing focus depth through the stack. Some approaches for intensity correction take the maxima or an average of the foreground of each image as a parameter of intensity and then apply an inverse function to compensate for intensity loss [12], [14], [19]; however, they are unsatisfactory when the background has some complexity. Other methods are time-consuming [20]–[25] or require complex acquisition [26]. Thus, presently there is no reliable, fast solution that can be used before 3D segmentation. Therefore, we apply 2D segmentation to each individual image and the resulting images are then processed using 3D techniques (Figure 2 and Figure 3). Cell borders are fuzzy, therefore thresholding is preferred to edge detection methods for segmentation. The method for defining the threshold *t* is critical and we have developed a binarisation method.

**Figure 4:**
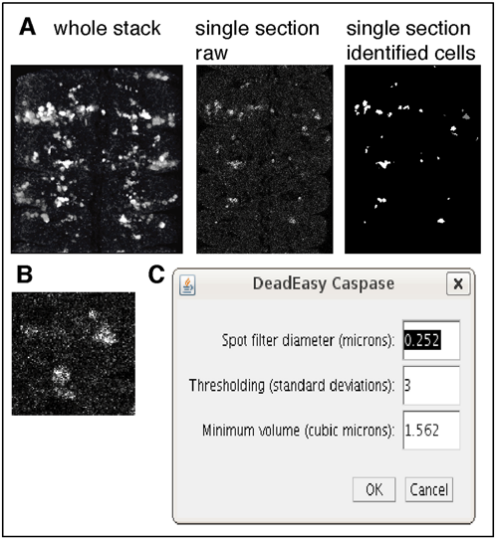
Qualities of Caspase stained cells identified and counted by DeadEasy. (A) Images showing a 3D rendering of the whole stack stained with Caspase, a single 0.25 µm section from this stack and the same section after object recognition by DeadEasy. (B) Enlarged image to show what Caspase stained cells look like, revealing the characteristic amorphous shape, low signal intensity and high background resulting from these antibodies. The accurate cellular parameters are given in Table 1. (C) User-friendly pop-window prompted by the programme to enable the user to modify the parameters easily, following the guidelines indicated in Table 3.

Embryos stained with Caspase3 yield images with several challenges and properties (Figure 4, Table 1). To overcome these problems, DeadEasy Caspase identifies cells based on a combination of pixel intensity and minimum volume in 3D (Figure 3). Due to fluorescence attenuation, Caspase-positive cells are more clearly seen in the first slices of the stack and for this reason it is not possible to use one threshold to binarise the whole stack; instead, a threshold value must be found for each image. The typical Caspase histogram *h(q)*, where *q* is the grey level intensity, of median-filtered Caspase images is composed of two modes, one corresponding to the background and another one to the embryo (Figure 3). A presumed third mode that would belong to the apoptotic cells is not visible due to the reduced number of pixels belonging to it. In some Caspase images, the histogram can become unimodal, if the image only includes the embryo and the background is so low as to disappear.

**Table 1:** Properties of cells counted by DeadEasy Caspase.

Method	Marker	Object shape	Object distribution	Minimum diameter µm	Minimum volume µm^3^	Signal	Background	Non-specific signal
**DeadEasy Caspase**	Anti-cleaved-Caspase-3: cytoplasmic	Irregular	Clustered	5.037	1.56	Weak	High & spotty	Can be

Given that there is not a mode corresponding to the Caspase stained cells, the following thresholding method was developed. First the histogram of the filtered image is obtained. The shape of the second mode, corresponding to the embryo, can be roughly approximated to a Gaussian function *G*(*q*) (Figure 3B), and the pixels belonging to the Caspase cells are considered outliers. A better approximation to shape of the mode can be obtained by mixing two Gaussians, which required, for instance the use of an Expectation-Maximisation procedure to find the function that best fix the shape of the mode [27]. However this solution is more complicated and it was found that approximating the mode to a simple Gaussian provided an optimum threshold. Since the Caspase histogram can be unimodal or bimodal, the embryonic mode is identified by finding the highest local maximum of the histogram. To identify the outliers, assuming the embryo's pixel grey level intensities are normally distributed, the Gaussian function *G_b_(q)* that best fits the shape of the embryonic mode is found. This is achieved by minimizing the square error between the histogram *h*(*q*) in the interval corresponding to the mode and *G*(*q*), that is:
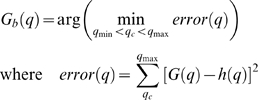
and 
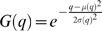
, *μ(q)* and *σ(q)* are the mean and standard deviation of the mode, calculated in the interval [*q*,*q*_max_], given by:
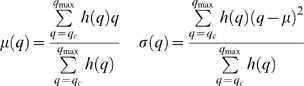

*q_c_* is a cut-off value given by the global minimum between the first and the second modes, when the histogram is bimodal, or the first local minimum of the histogram, when it is unimodal, and 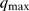 is the maximum grey level of the histogram. The threshold is obtained from the standard score (z-score), which rejects the outliers of the Gaussian function. The z-score is given by 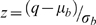 where 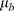 and 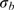 are the mean and standard deviation of the best Gaussian function respectively and *q* is the pixel intensity. It is considered that a grey level is an outlier if 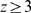, therefore the threshold *t* is given by 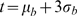.

Some raw images have small spots of high intensity, which can be confused with cells in later steps of the process (Figure 3C and Figure 4B). To eliminate these spots without affecting the threshold technique (if the spot filter is applied before the thresholding procedure the histogram is modified affecting the result), the raw images are filtered in parallel and the result is combined with the threshold outcome (Figure 3D and 3E). If a square window of side greater than the diameter of a typical spot, but smaller than the diameter of a cell, is centred in a cell, the mean of the pixel intensities inside the window should be close to the value of the central pixel. If the window is centred in a spot, the pixel mean should be considerably lower than the intensity of the central pixel. To eliminate the spots, a mobile window *W* is centred in each pixel. Let *p*(*x*,*y*) and *s*(*x*,*y*) be the original input image and the resulting filtered image respectively, and *m*(*x*,*y*) the average of the intensities inside the window centred in (*x*,*y*). If *m*(*x*,*y*) is lower than a certain proportion *α* with respect to the central pixel, it becomes black, otherwise it retains its intensity. That is:
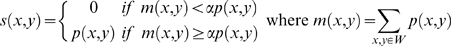
After thresholding, cells and small spots appear in white, while after spot filtering the spots appear in black. The result from both images is combined by using the following expression (Figure 3D and 3E):
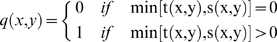



The combination of filtering and thresholding results in separating candidate objects (Caspase-positive cells) from background. The spot filter also separates cells that appear very close in the z axis.

To render the Caspase-positive cells more similar in appearance to the original raw images, three-dimensional morphological operations were performed throughout the whole stack (Figure 3D and 3E). Firstly, morphological closing followed by opening employing 6-connectivity is applied to further remove noise and to refine the candidate structures. Secondly, the objects containing holes are filled. Thirdly, a 2D watershed algorithm is used to separate cells that still appear connected. The final step is classification, whereby cells are identified and counted. Dying cells are irregular in shape and can only be identified by their volume. So a 3D labelling method using 18-connectivity is employed to identify each object of the stack. The number of voxels of each object is counted and thus the volume is obtained. Cells are identified from the candidate objects according to a minimum volume established empirically of 1.56 µm^3^, which corresponds to 100 voxels.

### Validation

**Figure 5:**
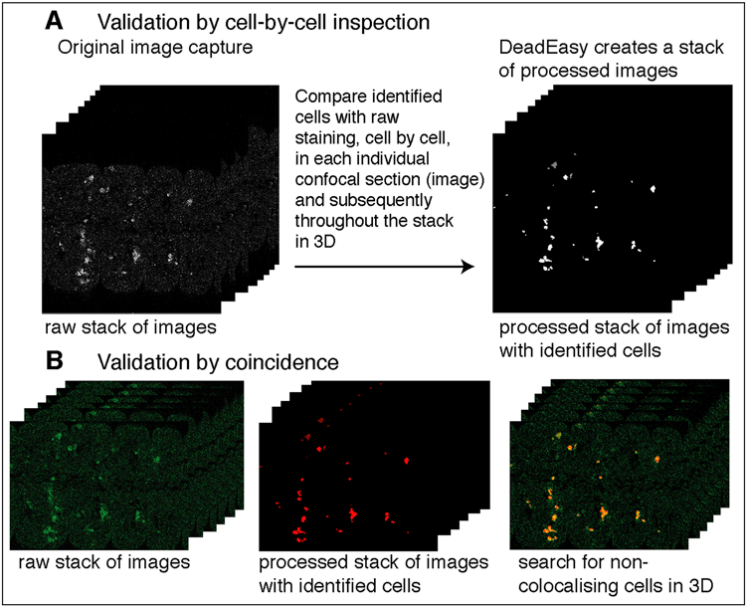
Validation of DeadEasy Caspase. (A) During processing, DeadEasy creates a second stack of confocal images reproducing the identified objects in locations that correspond to those of cells in the original raw stack. By placing the mouse over each of the objects, an identifying number is revealed (as shown in Figure 2), showing whether a cell is counted appropriately. We compared one cell at a time in this way through multiple stacks. (B) Using a second validation method, a merged stack can be created from the raw images (green) and the processed images with the identified objects (red). Colocalising cells in the merged stack (yellow) indicate the identified cells, green cells are false negatives and red cells are false positives.

Validation of DeadEasy Caspase was done in three complementary ways. Firstly, we tried comparing manual counting with automatic counting. However, manual counting was found to be unreliable (data not shown). Secondly, when running DeadEasy, a mirror-stack is produced per specimen, comprised of all the processed images with the identified objects (Figure 2 and Figure 5A). We compare the processed stacks with the raw stacks of images, looking at each individual cell, one confocal section at a time (i.e. not in projections). Each identified cell is labelled by DeadEasy with a number (an identifier): by placing the mouse over each of the cells the number comes up at the top of the image, and we can test one by one whether cells have been counted once, twice or more (or missed). Thirdly, validation was confirmed by creating a merged stack that combines the raw stack (e.g. green) and the processed stack (e.g. red) (Figure 5B): co-detected cells appear yellow, false negatives and false negatives appear as single colours. The merged stack can be checked by comparing it with each opened single channel in grey-scale (Figure 5B).

A low incidence of false positives (counted objects that were not stained cells or a cell that is counted twice) and false negatives (missed stained cells or two cells counted as one) was found (Table 2). Sensitivity is the probability of identifying an object as positive when it is in fact positive [given by true positives/(true positives plus false negatives), maximum value being 1], and it is high.

**Table 2:** Validation of DeadEasy Caspase programme.

Method	Validated for	Stacks	Cells	Real counts	False+(%)	False−(%)	Sensi-tivity
**DeadEasy Caspase**	embryos	21	1954	1973	20 (1.1)	39 (1.9)	0.98

**Key**: *Validated for*: indicates the antibody stainings and the stage in development of the samples used for validation. *Stacks*: number of stacks of images used for validation, each stack has 100–150 images. *Cells* : counted cells. *Real counts* : after verification. *Sensitivity*: maximum 1.

### Immunohistochemistry and image acquisition

*Drosophila* embryos were fixed and stained with rabbit anti-Caspase3 (at 1∶50 in embryos; Upstate Biotechnology) to visualise apoptotic cells. Antibody stained cells were detected with anti-Rabbit secondary antibodies, as appropriate, directly conjugated to the fluorochrome Alexa-488. Embryos were mounted in Vectashield (Vector Labs). Mounted whole embryos were scanned using a Radiance 2000 laser scanning confocal microscope. DeadEasy will work regardless of the confocal set up used as long as acquisition and processing are with the recommended parameters. The settings at the confocal microscope need to be fixed for all samples in each experiment in order for results to be comparable. In all cases, acquisition has to be set ensuring that the dynamic range of the histogram covers all grey values (i.e. from 0 to 255). The conditions for scanning are as follows: stacks were captured using 60× magnification oil immersion objective and zoom 1.6, laser excitation at 488 nm, acquisition resolution of 512×512 pixels, step between sections of 0.25 µm, 166 lines per second and no averaging/Kalman. This resolution results in a cubic voxel size of 0.25 µm^3^ (which can be checked on the pixel and voxel definitions given by the confocal software). DeadEasy Caspase will only work on cubic voxels. Fixed iris (pinhole), laser intensity, gain and offset are maintained throughout all samples of the same experiment. Laser life needs to be calibrated if experiment is carried out through an extended period. The quality of Caspase stainings changes with each batch. When comparing multiple genotypes, all experiments need to be carried out with the same batch of Caspase.

To achieve the specified accuracy, several points need to be taken into account. Firstly, stainings that produce non-specific signal (e.g. background fuzziness, tissue folds, edges) produce artefacts (examples in Figure 3F). Therefore, stainings need to be optimised, and badly stained samples discarded. Secondly, signal that falls below the specified intensity threshold will not be detected, and therefore weakly stained cells may be missed. Thirdly, Caspase stainings can frequently yield high, spotty background. DeadEasy will not identify Caspase stained cells if the background is very spotty (Figure 3F). This problem is overcome by optimising the staining conditions as follows. First, embryos are stained in very low numbers (about 5–10 µl worth of embryos) at a time, in 50 µl of Caspase. Anti-Caspase is used only once. Second, in double labellings, such as when needing to identify a balancer carrying a *lacZ* reporter, anti-βgal antibodies must be applied after anti-Caspase. We have found chicken anti-βgal (1∶100 and visualised with anti-Chicken Alexa 660) to give the best results. Third, no amplification - such as with Avidin, Streptavidin or a sequence of multiple secondary antibodies – is used, as this increases the background. Fourth, after scanning, samples are run through the programme to test if quantification is reliable. When the background is too high or signal too low, or there are obvious aberrations in the detection – such as extended areas (e.g. tissue folds) that do not correspond to Caspase stained cells (Figure 3F) - these samples are discarded as the experiment did not work. Fifth, even in excellent stainings there can be sources of false positives. These may be non-specific staining at embryonic edges or tissue folds; small, intense spots along axons; and auto-fluorescence from the amnioserosa and/or gut cells. If there are only a small number of such false positives they must be eliminated either manually or by creating a separate stack comprised only of the affected slices and tracing an ROI around the false positives. The ROI can then filled black and the programme run again for counting using DeadEasy Count. Care must be taken to avoid double counting when merging the results form two or more sub-stacks.

### Software development, use, and further adaptations

The algorithm was developed using Java, as a plug-in for the freely available software ImageJ, in a Pentium 4, 3 GHz computer with 1.5 GB RAM. Before installing the DeadEasy plug-in, ImageJ needs to be installed from the internet. ImageJ is a public domain image-processing program, developed in Java and written by Wayne Rasband. The most recent version can be downloaded from the ImageJ web-site (http://rsb.info.nih.gov/ij/). Once ImageJ is installed, copy the DeadEasy Caspase plug-in to the ImageJ plugins folder and restart ImageJ. The DeadEasy Caspase plug-in can be downloaded either via the ImageJ website or from our group's web-page (www.biosciences.bham.ac.uk/labs/hidalgo). To run the programme, open a confocal stack through ImageJ, go to Plug-ins, select DeadEasy Caspase plug-in and simply run. To run multiple stacks in one go, collect all the stacks into one folder and run Lots-DeadEasy Caspase.

To achieve accuracy, the voxel size must be defined (automatically or manually) prior to automatic counting in “Properties” within ImageJ. To count cells within a ROI, the ROI must be adjusted to each specimen for greater accuracy based on anatomical landmarks, since not all individuals are of identical size and variations with fixation can also affect size. DeadEasy was developed for Drosophila, but it can be used to count cells in different organisms, as long as cell size, type and quality of images are comparable, scanning conditions are set as recommended and voxel sizes are defined. The parameters to detect the objects can be changed to suit other samples, using a user-friendly window (Figure 4C). The consequences of altering the parameters from our default settings are given in Table 3.

**Table 3:** Parameters that can be altered by the user and the consequences.

DeadEasy Caspase in Embryos	
Parameter	Consequences of parameter change in cell count
**Spot diameter**	This is to eliminate noise (i.e. spots) in 2D. Increasing the diameter will decrease the current cell count because more particles will be considered as noise and they will be eliminated. For a different sample type, the diameter could be increased if the size of the Caspase stained cells increases compared to the default settings.
**Thresholding**	This is to separate signal from noise. Increasing the standard deviation away from the mean value of the Gaussian function will decrease the number of pixels considered to belong to the cells. This can be set empirically based on the quality of the background and the intensity of the signal.
**Minimum volume**	This is to detect the objects in 3D. Increasing the minimum volume will decrease the current count, as more small particles will be excluded. For a different sample type, the minimum volume could be increased if the size of the Caspase stained cells increases compared to our default settings.

This table sows the parameters that the user can modify after accessing through ImageJ theprogrammes written in Java. Any parameter modification will require cycles of validation and further modification by the user, until the desired accuracy for the sample and staining in use is reached.

### Counting of double-labelled cells

DeadEasy can be used to count cells stained with Caspase and another different antibody. To count the subset of cells stained with two antibodies, run first DeadEasy Caspase; then compare the stack of images containing the results of DeadEasy with the raw stack of images from the second label using the “Calculations with AND” function in ImageJ. This function creates a new stack of images containing only the cells that have both labels. These colocalising cells can now be counted again using the ImageJ macro DeadEasy Count.

### Genetics

The stock used as wild-type was *y w*. Mutants: *H99/TM6BlacZ*. Mutant embryos were identified by the absence of anti-βgal signal when staining the embryonic population with anti-βgal antibodies, and their head-involution defect.

## Results and Discussion

The number of apoptotic cells in the central nervous system can be phenomenal (Figure 1). DeadEasy Caspase is an image processing and pattern recognition algorithm that identifies objects (anti-cleaved-Caspase-3 stained cells) over background (non-specific antibody staining) from a collection of images that make up the complete 3D structure of the ventral nerve cord (VNC), and counts the objects automatically (Figure 2).

DeadEasy Caspase counts dying (apoptotic) cells stained with anti-cleaved-Caspase-3 (hereafter called Caspase) (Figure 2 and Figure 3), a widely used marker for apoptotic cells, in Drosophila embryos. Caspase is initially cytoplasmic and as apoptosis progresses it reveals intense, shrunken cells. Embryos stained with Caspase yield images with the following characteristics (Figure 4, Table 1): (1) Caspase-positive cells have irregular shape and size. (2) There is high non-specific background of uniformly distributed small spots. (3) Florescence attenuation with increasing depth is pronounced. (4) Signal intensity is low. DeadEasy Caspase has been validated to count automatically the number of dying cells in the Drosophila embryo and for this task it is very accurate. The characteristics of the marker (Table 1) and the resulting images (Figure 4) can be used as a guide to estimate whether DeadEasy could be used for other samples (e.g. non-Drosophila samples). DeadEasy Caspase detects apoptotic cells accurately in Drosophila embryos (Table 2), but not in larvae due to the intense non-specific staining of axons. The programme (written in Java) is freely available through ImageJ, and the source code, the parameters indicated in Table 1, Table 3, and in the diagrammatic algorithm, can be modified by the user to count apoptotic cells in a different sample of choice. A user-friendly interface has been included with the programme to enable easy modification of the parameters (Figure 4C). However, upon parameter modification the accuracy of DeadEasy may be compromised and any parameter change will need to be validated by the user (Table 3).

DeadEasy Caspase can also be used to count automatically the subset of cells stained with another overlapping marker. This is done by combining the results of DeadEasy and the other marker in ImageJ using the “Calculations” menu, and counting the result automatically again with our purposely written DeadEasy Count macro (obtained also through ImageJ).

To measure the accuracy of DeadEasy Caspase, cells were counted automatically and the resulting cells were verified one by one (Figure 5). A low incidence of false positives and false negatives, while high sensitivity, were found (Table 2). The sources of false negatives are low signal intensity (i.e. objects obvious to the eye are below threshold), or cells are too close to each other and appear joined in at least one image or slice (e.g. two cells counted as one). The main source of false positives are background spots (i.e. unspecific spots counted as cells, see Table 3) or folds in the tissue (Figure 3F). DeadEasy results are reproducible, while human manual counting is not. It performs consistently as it always yields the same cell number count for a given sample regardless of the number of times it is counted. Repeated human counting by the same or different persons of a large number of cells invariably results in a slightly different count. This means that with DeadEasy, constant and objective criteria are used to compare multiple genotypes and multiple samples.

DeadEasy Caspase is very fast: (1) it has been designed to use the shortest possible scanning time at the confocal microscope, by eliminating any averaging noise reduction step. A standard embryonic scan of the entire nerve cord (approximately 150 slices) takes about 10 minutes. (2) DeadEasy programmes count cells in about 1 minute per animal, for each confocal stack of around 150 slices. By comparison, manual counting of only a subset of Caspase stained cells in the embryonic VNC takes around one week per genotype [28]. A very large number of samples can be loaded in one go (e.g. 400 confocal stacks or more) to be quantified automatically over-night. The reduction in workload by using DeadEasy is phenomenal. Across genotypes comparisons of cell number is not possible without DeadEasy. DeadEasy enables quantitative analyses unfeasible otherwise.

**Figure 6:**
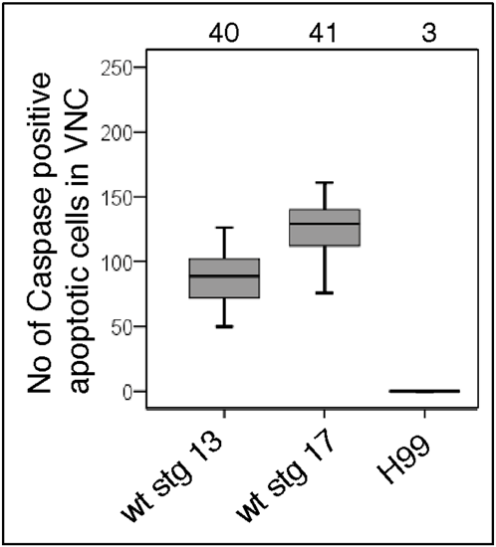
Examples of application of DeadEasy Caspase to address biological questions. DeadEasy Caspase is used to count the number of apoptotic cells stained with Caspase in wild-type embryos at different stages and in H99 mutants lacking apoptosis. Numbers over box-plots indicate number of embryos analysed per genotype.

To test the value of DeadEasy software for addressing biological questions we carried out temporal profiles of dying cells, in wild-type and H99 mutant embryos (lacking apoptosis). We show here in box-plots that the number of apoptotic neurons increases between stages 13 and 17 in Drosophila embryos (Figure 6), consistently with a previous report of manual quantification of Caspase stained cells [11]. As control for DeadEasy Caspase, H99 embryos deficient in apoptosis were analysed, where Caspase staining is only non-specific. DeadEasy Caspase did not detect any objects in H99 mutant embryos stained with Caspase (Figure 6). The resulting distributions in apoptotic cell number are spread out (reflected by the box-plot distribution), reflecting variability in cell number across a population. This variability is not due to variable cell counting by DeadEasy, but to: (1) Slight variations in the cells included within the ROI used for cell counting. (2) Variations in staining quality in separate experiments. (3) Subjective estimation of the age of the embryo. (4) Confocal laser life decay over time, that however becomes negligible if appropriate care of the laser is taken. (5) Biological origin: the number of dying cells is not cell lineage-invariant across cell types in Drosophila.

DeadEasy can be used to compare cell counts between large samples of wild-type and mutant specimens, to infer gene function. The power of DeadEasy Caspase to address biological questions has already been demonstrated in two publications. We have previously used DeadEasy Caspase to analyse trophic support functions of PVF signalling [29] — the Drosophila homologues of PDGF — and to provide functional evidence of the existence of a Drosophila neurotrophin family [28]. The latter shows that over-expression of Drosophila neurotrophins (DNTs) in neurons results in a reduction in apoptosis compared to wild-type, whereas mutations in DNTs result in an increase in apoptosis. These findings demonstrate that neurotrophin functions are conserved throughout evolution and they support the notion of a common molecular origin for the brain throughout the animals [28]. This analysis would have been unfeasible without DeadEasy.

To conclude, we have developed a method for the automatic quantification of apoptotic cell number in intact Drosophila embryos. For the direct use as specified (i.e. Drosophila embryos) running DeadEasy Caspase is extremely easy: open “ImageJ”, choose “Plug-ins” from menu, scroll down, run “DeadEasy Caspase”. DeadEasy Caspase is extremely fast, and it eliminates the tedium of manual cell counting. Because automatic counting is objective, reliable and reproducible, comparison of apoptotic cell number between specimens and across genotypes is considerably more accurate with DeadEasy than with manual counting. Since automatic counting is so fast, large sample sizes can be handled. DeadEasy Caspase can also be used to count subsets of cells stained with two different markers for co-detection studies.

DeadEasy Caspase could be used for automatic counting of apoptotic cell number in other tissues or model organisms (e.g. fish, mouse) so long as stainings of comparable qualities are used to visualise cells of comparable sizes, as indicated in Table 1 and Table 3 and Figure 4. In such cases, users should optimise their stainings first to obtain image qualities of the defined specifications and show go through rounds of improvement (changing the parameters as shown in Table 3) and validation against their own data to achieve the desired accuracy. DeadEasy Caspase has been written as a freely available ImageJ plug-in. We have made the programme and source code available to users through ImageJ, who can creatively change the parameters either in Java or using the user-friendly pop-up window that we provide, to suit, optimise and validate the programme relative to their samples. DeadEasy Caspase will be of interest for Drosophila researchers and for the broader scientific and biomedical communities.
